# *PdStuA* Is a Key Transcription Factor Controlling Sporulation, Hydrophobicity, and Stress Tolerance in *Penicillium digitatum*

**DOI:** 10.3390/jof9090941

**Published:** 2023-09-18

**Authors:** Yujie Du, Jinfan Zhu, Zhonghuan Tian, Chaoan Long

**Affiliations:** 1 National Key Laboratory for Germplasm Innovation & Utilization of Horticultural Crops, National R&D Center for Citrus Preservation, National Centre of Citrus Breeding, Huazhong Agricultural University, Wuhan 430070, China; yeast427@163.com (Y.D.); a616433700@163.com (J.Z.); 2 Shenzhen Institute of Nutrition and Health, Huazhong Agricultural University, Wuhan 430070, China; 3 Shenzhen Branch, Guangdong Laboratory for Lingnan Modern Agriculture, Genome Analysis Laboratory of the Ministry of Agriculture, Agricultural Genomics Institute at Shenzhen, Chinese Academy of Agricultural Sciences, Shenzhen 518000, China

**Keywords:** *Penicillium digitatum*, conidia, conidiophore, *PdStuA*, stress tolerance, hydrophobicity

## Abstract

*Penicillium digitatum* has become one of the main pathogens in citrus due to its high spore production and easy spread. In this study, the function of the APSES transcription factor *StuA* in *P. digitatum* was characterized, and the results indicated that it was involved in conidium and conidiophore development. No conidiophores were observed in the mycelium of the ∆*PdStuA* mutant that had grown for two days, while an abnormal conidiophore was found after another two days of incubation, and only small thin phialides as well as a very small number of spores were formed at the top of the hyphae. Moreover, it was observed that the ∆*PdStuA* mutant showed various defects, such as reduced hydrophobicity and decreased tolerance to cell wall inhibitors and H_2_O_2_. Compared to the original *P. digitatum*, the colony diameter of the ∆*PdStuA* mutant was not significantly affected, but the growth of aerial hyphae was obviously induced. In in vivo experiments, the spore production of the ∆*PdStuA* mutant grown on citrus fruit was remarkably decreased; however, there was no significant difference in the lesion diameter between the mutant and original strain. It could be inferred that less spore production might result in reduced spread in citrus, thereby reducing the green mold infection in citrus fruit during storage. This study provided a gene, *PdStuA*, which played key role in the sporulation of *P. digitatum*, and the results might provide a reference for the molecular mechanisms of sporulation in *P. digitatum*.

## 1. Introduction

Citrus fruit is greatly adored by people both locally and abroad because of its superior nutritional value, flavor, and taste [1,2]. However, citrus fruit is prone to decay due to infection during harvesting, transportation, and storage [3]. Infectious pathogens (such as *Penicillium digitatum*, *Geotrichum citri-aurantii*, *P. italicum*, and *Colletotrichum gloeosporioides*) have had a significant negative impact on the development of the citrus industry [2,4]. Under severe conditions, it might exceed 50% and cause significant economic losses as well as have an impact on the market supply and export sales [1,5]. Spores of infectious pathogens could swiftly disperse under the right environmental conditions, germinate, and proliferate at citrus wounds. A significant number of spores can then form on the citrus fruit’s surface, which can spread and infect other citrus fruits, resulting in worse green mold [5]. Therefore, regulating the prevalence of green mold depends greatly on knowing the process of *P. digitatum* spore generation. Understanding the mechanisms involved in the production of conidia and their germination is crucial.

Conidiation is the most prevalent reproductive mechanism for many filamentous fungi [6]. Each filamentous fungal colony could produce a sizable number of spores, which subsequently disperse via the air to other locations and aggravate the disease incidence. The spores’ central regulatory gene cluster *BrlA*-*AbaA*-*WetA* has been shown to control spore production in *Aspergillus*, *Neurospora crassa*, and *P. digitatum* in several studies [6,7,8]. Previous research has found that deletion of the *PdBrlA* gene inhibits conidiophore generation, prompting mycelia to grow aerially and eventually form colonies resembling white cotton [6]. The *PdAbaA* gene regulates conidiophore formation but lacks a differentiation function, resulting in the inability to produce spores [6]. The *PdWetA* gene did not affect the development of the conidiophore or spore production but inhibited the production of spore pigments and produced spore morphology abnormalities, resulting in white colonies [6]. Additionally, only *PdLaeA* and spore pigment-related genes have been investigated thus far [9,10]. In contrast, the *LaeA* gene could bind with the velvet protein family to regulate spore production, and the loss of the *PdLaeA* gene caused a doubling of conidium production [9]. The absence of pigment-related genes in conidia only affected the accumulation of pigment in the spores and did not affect conidial production [10]. Other sporulation-related genes have not been reported.

The APSES protein family is a class of transcription factors unique to fungi, including Asm1, Phd1, Sok1, Efg1, and StuA. StuA is involved in various biological functions, including conidiophore formation, spore generation, hyphal growth, secondary metabolism, and pathogenicity [11,12,13,14,15]. Although the StuA protein is highly conserved across a variety of fungi, its potential functions are not consistent. For example, in *Fusarium graminearum*, the perithecia and sexual ascospores were completely unable to form but could generate spores from the hyphae [16]. Ascospore and cleistothecium development were difficult when the *StuA* gene was deleted in *Aspergillus nidulans* [17,18]. In *F. oxysporum*, the *StuA* gene could stimulate the growth of macroconidia while inhibiting the formation of chlamydospores and had no effect on the development of microconidia [19]. In *F. verticillioides*, the absence of the *StuA* gene almost completely impaired the ability to produce macroconidia and reduced microconidial production [13]. The pathogenicity of the *StuA* gene depends on the species. Pathogenicity declined in *F. graminearum*, *Arthrobotrys oligospora*, *Leptosphaeria maculans*, and *Zymoseptoria tritici*, whereas the *StuA* gene had an optional function in *F. oxysporum* [15,16,19,20,21]. The role of *StuA*, however, was consistent in terms of pigment production and preventing pigment deposition [14,21,22].

We identified the *StuA* gene in *P. digitatum* and investigated its function to better understand the sporulation process of *P. digitatum*. In this study, the phylogenetic relationships of the StuA protein in *P. digitatum* among some different filamentous fungi were characterized, and the function of the *StuA* gene in spore development and conidiophore formation in *P. digitatum* were also determined. In addition, the responses of *P. digitatum* to Calcofluor White (CFW), Conger red (CR), sodium dodecyl sulfate (SDS), and H_2_O_2_ were determined after *StuA* gene deletion, and the pathogenicity was also observed. Our research results provide new insights into the spore production process of *P. digitatum*.

## 2. Materials and Methods

### 2.1. Fungal Strains and Culture Conditions

In this investigation, a wild-type strain of *P. digitatum* N1 was employed. All the fungi were cultured on potato dextrose agar (PDA, Coolaber, Beijing, China) plates. Conidia were harvested from a five-day PDA plate. Without any specific instructions, a hemocytometer was used to set the spore concentration at 1 × 10^6^ CFU mL^−1^. The entire experiment was carried out three times independently, with three replicates.

### 2.2. Targeted Gene Disruption and Complementation

*PdStuA*-UF/R and *PdStuA*-DF/R primers were used to amplify the *P. digitatum* genome, as described in previous work, to produce upstream and downstream fragments of the *PdStuA* gene [10]. The recombinant vectors were pCHPH and pCNEO, which were constructed by Cheng et al. [23] and kindly given by the authors; the vectors were preserved in our lab. These segments were fused with pCHPH to create a knockout vector. Next, pCNEO was coupled with the full *PdStuA* gene (including promoter and terminator fragments) to create a complementary vector. The ∆*PdStuA*, *PdStuA*^C1^, and *PdStuA*^C2^ (*PdStuA*^C1-2^) mutants were identified by PCR and RT-qPCR. The required primers are shown in Table S1.

### 2.3. Phylogenetic Analysis

NCBI tools were utilized to examine the StuA protein domain (https://www.ncbi.nlm.nih.gov/Structure/cdd/wrpsb.cgi, accessed on 20 July 2023) based on techniques employed in other studies [24]. The StuA proteins of 13 different fungi were then obtained from the NCBI database and analyzed with MEGA7. This tree has 1000 repeated bootstrap analyses using the neighbor-joining method.

### 2.4. Phenotypic Analysis and Subcellular Organelle Observation

On 60 mm PDA plates, the N1, ∆*PdStuA*, and *PdStuA*^C1-2^ strains were grown for 5 d. The colony diameter and morphology of the four strains were determined. Spore production was statistically evaluated after distilled water was placed over each strain, as previously described [9]. 

We used a prior approach [25], in which 2.5 μL of the spore suspension (1 × 10^6^ CFU mL^−1^) was dropped onto the PDA medium and covered with a cover glass to assess the formation of conidiophores. Images were taken under an optical microscope after 2 and 4 d.

We tested whether the *PdStuA* gene contributed to surface hydrophobicity by adding 10 μL of various solutions (sterile distilled water, 0.2% gelatin (Hushi, Shanghai, China), 250 mg mL^−1^ Tween 20 (Hushi, Shanghai, China), and 0.02% SDS (Hushi, Shanghai, China) + 0.5 mM EDTA (Hushi, Shanghai, China)) [26]. After 12 h, the surface hydrophobicity of the N1, ∆*PdStuA*, and *PdStuA*^C1-2^ strains was assessed. Three plates with 10 water droplets were used for each strain, and the entire experiment included three biological replicates.

We employed scanning electron microscopy (SEM) and transmission electron microscopy (TEM) to analyze the conidiophores and subcellular organelles of the N1 and ∆*PdStuA* strains. As mentioned earlier [4], 100 μL of the spore suspension was coated on the PDA plates. The mycelia were collected on Days 2 and 4. The samples observed using TEM were handled according to a prior approach [27].

### 2.5. Stress Tolerance Assays

A PDA plate containing the *P. digitatum* spore suspension (1 × 10^6^ CFU mL^−1^) was perforated into a 5 mm uniform cake using the previous method with a small modification [28]. It was placed on PDA plates with 1 mM H_2_O_2_ (Hushi, Shanghai, China), 1 mg L^−1^ CFW (Sigma, St. Louis, MO, USA), 10 mg L^−1^ CR (Hushi, Shanghai, China), and 5 mg L^−1^ SDS (Hushi, Shanghai, China), respectively. The diameter of each strain was then measured to determine the rate of inhibition under various chemical stressors on Day 5. The inhibition rate = (colony diameter of each strain on PDA—colony diameter on stressor)/the colony diameter of each strain on PDA × 100%. These tests were carried out three times.

### 2.6. Virulence Assays

Satsuma mandarin fruit from the same batch, mature and undamaged, was chosen. The citrus was soaked for 2 min in 2% sodium hypochlorite solution and then rinsed twice with distilled water. Subsequently, the citrus was air-dried on a clean bench before use. Two wounds (5 × 5 × 2 mm) were created using a sterile wounding tool and were evenly distributed on the citrus’s equatorial line [29]. Each wound was inoculated with 10 μL of a spore suspension (N1, *PdStuA*, and *PdStuA*^C1-2^), and the infected citrus was cultured for 4 d. The fruits were placed in a 25 °C container at a relative humidity of ≥98%. The entire experiment was repeated three times, with 10 fruits in each strain.

### 2.7. Quantitative Real-Time PCR Assays (RT-qPCR)

Based on previous methods [4,29], hyphae from N1 and Δ*PdStuA* mutants were gathered and frozen in liquid nitrogen after incubation for 2 d. TRIzol was used to isolate *P. digitatum* RNA. qPCR SYBR Green Master Mix (Vazyme, Nanjing, China) was used to prepare the reaction. The *ACTIN* gene was used as a reference gene in this study. The relative expression level (REL) was calculated using the method described according to a prior approach [30], and the formula was 2^−ΔΔCt^. Next, we selected some genes to confirm the outcomes of the aforementioned experiment. Genes such as *PdRodA* and *PdSom1*, which are involved in sporulation, as well as central regulatory pathway genes of conidiation (*PdBrlA*, *PdAbaA*, and *PdWetA*) were chosen. The relevant primers are listed in Table S2.

### 2.8. Statistical Analysis

In this experiment, the significance differences were determined using SAS software (version 8.1; SAS; Institute, Inc., Cary, NC, USA). The statistical significance was assessed using Duncan’s multiple range test. Significant changes were indicated by different letters or * (*p* < 0.05). Image production was performed with GraphPad Prism 8.0 (GraphPad Software, San Diego, CA, USA).

## 3. Results

### 3.1. Summary of PdStuA in P. digitatum

**Figure 1:**
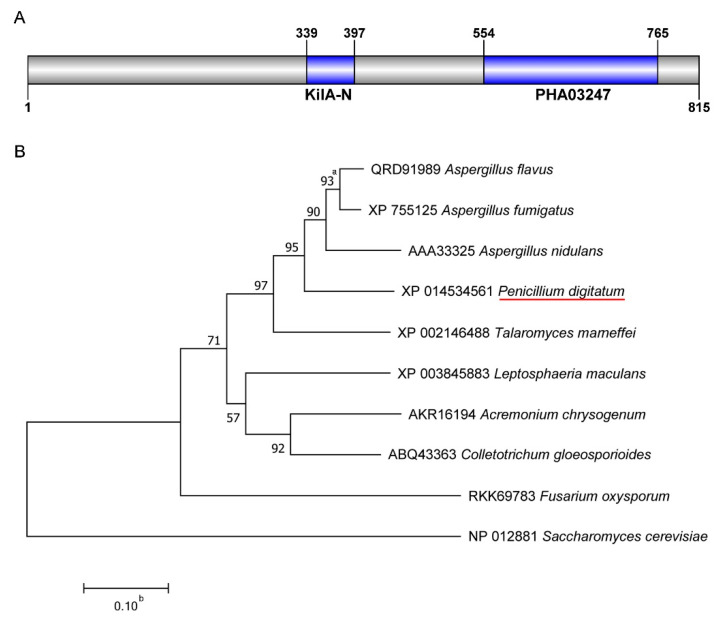
Domain and phylogenetic tree analysis of StuA protein. (**A**) The conserved domain of the StuA protein in *P. digitatum* was retrieved from NCBI (https://www.ncbi.nlm.nih.gov/Structure/cdd/wrpsb.cgi, accessed on 20 July 2023). (**B**) Phylogenetic analysis of StuA proteins was performed using MEGA7 by the NJ (neighbor-joining) method. a, The number represents the support value, and it is used to represent the reliability of the branch structure. b, The number represents the distance scale. The red underline represents StuA in *P. digitatum*.

We discovered a potential APSES transcription factor, StuA, according to the annotation of the *P. digitatum* genome. The *PdStuA* gene (PDIP_46840) had a total length of 3111 bp and an open reading frame of 2448 bp. According to the NCBI domain analysis, the total length of the PdStuA protein was 815 amino acids (AA), including the KilA-N domain (a conserved DNA-binding domain) located between 339 AA and 397 AA and the PHA03247 domain (a large tegument protein) located at 554 AA and 765 AA (Figure 1A). The StuA protein sequences from different fungi and PdStuA protein were used for phylogenetic analysis. The PdStuA protein of *P. digitatum* was deduced from the phylogenetic tree to be more closely linked to homologs in *A. flavus*, *A. fumigatus*, and *A. nidulans*. The homolog in *S. cerevisiae* was the most different from the PdStuA protein (Figure 1B).

### 3.2. Construction and Verification of PdStuA-Deletion and PdStuA-Complementation Mutants

**Figure 2:**
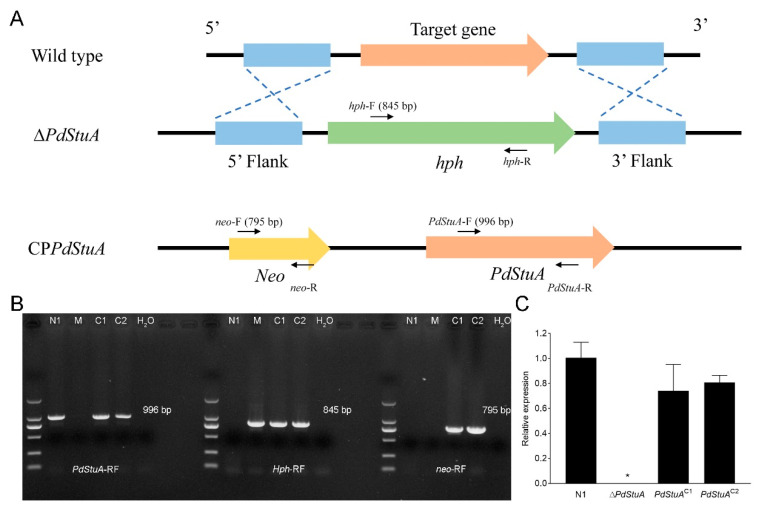
Identification and deletion of the *PdStuA* gene in *Penicillium digitatum*. (**A**) Schematic diagram of ∆*PdStuA* and *PdStuA*^C1-2^ production. (**B**) PCR identification of N1, ∆*PdStuA*, *PdStuA*^C1^, and *PdStuA*^C2^ strains. (**C**) The gene expression of *PdStuA* was verified through RT-qPCR. N1: wild-type strain; M: ∆*PdStuA* mutant; C1: *PdStuA*^C1^ mutant; C2: *PdStuA*^C2^ mutant. H_2_O was used as a negative control. * Indicates significant differences (*p* < 0.05).

Using the homologous recombination method, we obtained ∆*PdStuA* and *PdStuA*^C1-2^ mutants (Figure 2A). *PdStuA*-RF, *Hph*-RF, and *neo*-RF primers were used for the N1, ∆*PdStuA*, and *PdStuA*^C1-2^ mutants, respectively. The PCR amplification products are shown in Figure 2B. The results showed that the *PdStuA* gene could not be amplified, but the *Hph* gene could be amplified in the ∆*PdStuA* mutant. In addition, we conducted RT-qPCR experiments on the four strains. The *qPdStuA* gene could not be amplified from only the ∆*PdStuA* mutant, and its expression was undetectable (Figure 2C). These results indicated that we successfully obtained the ∆*PdStuA* and *PdStuA*^C1-2^ mutants.

### 3.3. PdStuA Affects Spore Production but Does Not Affect Hyphal Development

**Figure 3:**
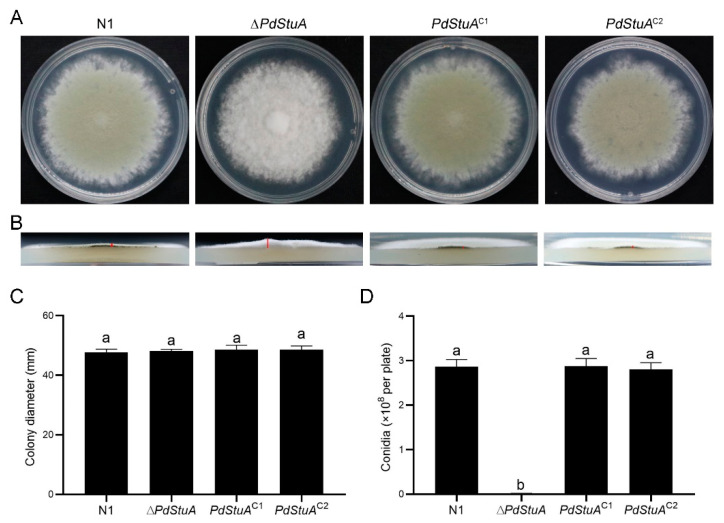
The role of *PdStuA* in the growth and spore production of *P. digitatum*. (**A**) Colony morphology of the N1, ∆*PdStuA* mutant, and *PdStuA*^C1-2^ strains on PDA medium on Day 5. (**B**) The side views of the four strains were analyzed for aerial hyphae and colony thickness. The red bar represents the thickness of the colony. (**C**) The growth diameter of the four strains was measured on Day 5. (**D**) The number of conidia in the four strains was measured on Day 5. Different letters indicate significant differences (*p* < 0.05).

As shown in Figure 3A, the growth morphology of the N1 and *PdStuA*^C1-2^ strains was similar, with colonies having a green appearance. The absence of the *PdStuA* gene led to the growth of aerial hyphae and the formation of white, thick, flocculent colonies (Figure 3B). The ∆*PdStuA* mutant colony was thicker than the N1 strain colony. However, there was no significant difference in the growth diameter of the four strains (Figure 3C). Additionally, the absence of the *PdStuA* gene significantly hindered the spore production of *P. digitatum*. There were 1.38 × 10^6^ spores on each ∆*PdStuA* strain plate, while the N1 strain had a spore count of 2.86 × 10^8^ per plate, a difference of 5 × 10^2^-fold. The phenotype of the *PdStuA*^C1-2^ strains was restored (Figure 3D).

### 3.4. PdStuA Controls the Occurrence of Conidiophores

**Figure 4:**
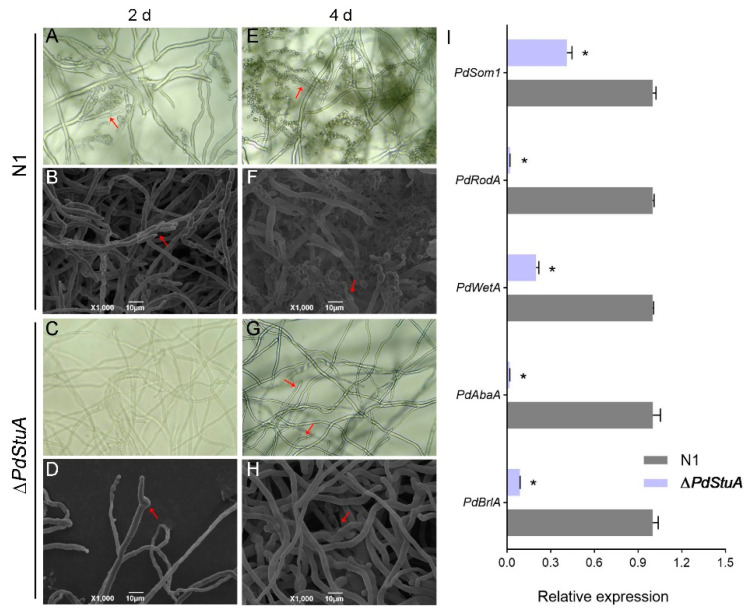
*PdStuA* plays an important role in the development of spores and conidiophores. (**A**–**D**) Microscopic and SEM images of conidiophore morphology on Day 2. (**E**–**H**) Microscopic and SEM graphs of conidiophore morphology on Day 4. Figures (**A**,**C**,**E**,**G**) were examined by optical microscope under ×400 magnification. The bar is shown in the figure. The red arrows represent conidiophores. (**I**) Relative expression analysis of sporulation-related genes. The experiment was conducted using three biological replicates. * represents significant differences (*p* < 0.05).

After growing on PDA plates for two days, complete conidiophores were detected in strain N1, and there were a lot of conidia. Apical swelling without any extra structure was visible in the SEM images. The N1 strain had more conidiophores and longer spore chains on Day 4. The only observation that could be made for the ∆*PdStuA* mutant was the expansive top hyphae and thin, immature, faulty, and tiny phialides. SEM images also revealed that the ∆*PdStuA* mutant had very low spore and conidiophore production capacities. Subsequently, the RT-qPCR validation of the genes involved in spore generation was carried out. The ∆*PdStuA* mutant had a dramatic decline in *PdbrlA*, *PdabaA*, *PdWetA*, *PdRodA*, and *PdSom1* when compared to the N1 strain, which was consistent with the outcomes of conidiophore development (Figure 4). Among them, the transcript levels of three genes (*PdbrlA*, *PdabaA*, and *PdRodA*) in the ∆*PdStuA* mutant decreased by 10-fold, 20-fold, and 20-fold, respectively.

### 3.5. PdStuA Is Involved in Hyphal Hydrophobicity in P. digitatum

**Figure 5:**
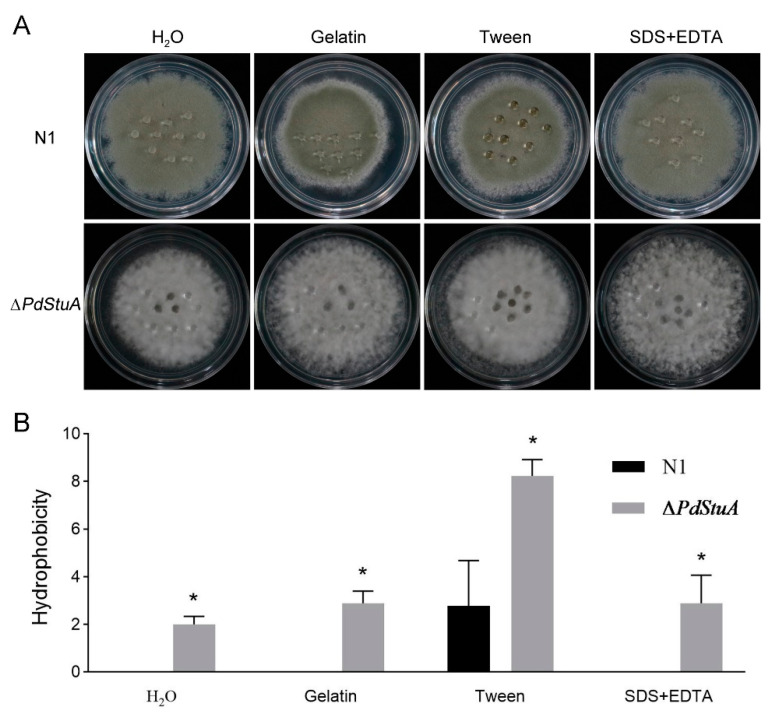
*PdStuA* is necessary for surface hydrophobicity. (**A**) The surface hydrophobicity of N1, Δ*PdStuA*, and *PdStuA*^C1-2^ strains was evaluated by adding 10 μL of sterile distilled water, 0.2% gelatin, 250 mg mL^−1^ Tween 20, and 0.02% SDS + 0.5 mM EDTA, respectively. Each fungal colony was dropped in 10 drops of solution, with three biological replicates. (**B**) The number of dissolved water droplets was used as the strength of hydrophobicity: the larger the number, the poorer the hydrophobicity. * indicates *p* < 0.05.

This study applied four different solutions (sterile distilled water, 0.2% gelatin, 250 mg mL^−1^ Tween 20, and 0.02% SDS + 0.5 mM EDTA) to colonies of *P. digitatum* to determine whether the *PdStuA* gene changed how hydrophobic the aerial hyphae were. According to the research results, after 12 h of treatment with H_2_O, gelatin, SDS, and EDTA, the N1 mycelium did not permeate. Owing to the function of Tween as a suspension aid, 2.78 droplets of Tween solution were sufficient to permeate the N1 mycelium. The permeation effect of the ∆*PdStuA* mutant was significantly higher than that of the N1 strain. Under treatment with the four solutions, 2, 2.89, 8.22, and 2.89 droplets permeated the ∆*PdStuA* mutant, respectively. It was interesting that only the center of the ∆*PdStuA* mutant colonies showed a depression, forming a “wettable” phenotype (Figure 5). The above results indicated that the *PdStuA* gene was involved in the hydrophobicity of mycelia.

### 3.6. PdStuA Takes Part in Cell Wall Integrity

**Figure 6:**
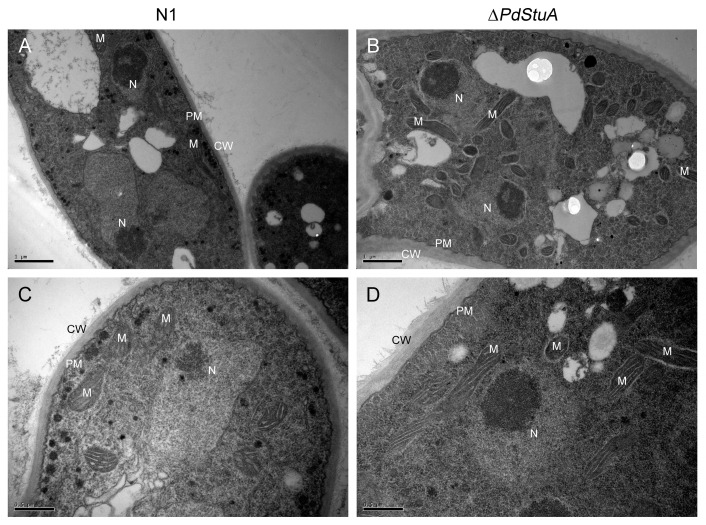
TEM micrographs of N1 and ∆*PdStuA* strains. (**A**,**C**) TEM micrographs of N1 strain at different magnifications. (**B**,**D**) TEM micrographs of ∆*PdStuA* mutant at different magnifications. The meanings of CW, PM, M, and N are cell wall, plasma membrane, mitochondrion, and nucleus, respectively. The scale is shown with a black bar at the left bottom in each figure.

TEM was employed for the ultrastructure observation in both the ∆*PdStuA* mutant and N1 strain. According to the research results, the N1 strain had clear nuclei, nucleoli, and a sizable number of mitochondria, all of which were arranged neatly. The Δ*PdStuA* mutant also had similar characteristics. Through TEM observation, the cell wall of ∆*PdStuA* was thicker than that of the N1 strain (Figure 6).

### 3.7. PdStuA Is Essential for Stress Responses

**Figure 7:**
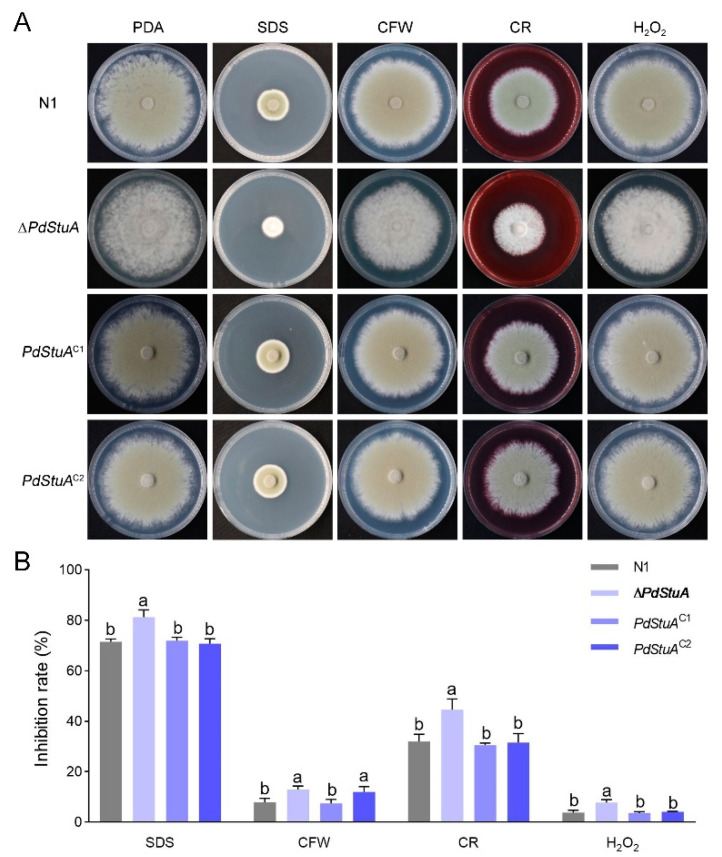
*PdStuA* is an important regulator of the stress response of *P. digitatum*. (**A**) Colony morphologies of N1, Δ*PdStuA*, and *PdStuA*^C1-2^ strains grown on PDA, 5 mg L^−1^ SDS, 1 mg L^−1^ CFW, 10 mg L^−1^ CR, and 1 mM H_2_O_2_ for 5 d. (**B**) The growth inhibition rate of *P. digitatum* under different stress agents. Columns with different letters are significantly different (*p* < 0.05).

We stressed *P. digitatum* with various stressors (CFW, SDS, CR, and H_2_O_2_) to determine whether the *PdStuA* gene was involved in the stress response. In comparison to the N1 strain, the Δ*PdStuA* mutant was more sensitive to CFW, SDS, CR, and H_2_O_2_. The phenotype was restored in *PdStuA*^C1-2^ mutants (Figure 7). The above results indicated that deletion of the *PdStuA* gene affected the cell wall integrity and oxidative stress.

### 3.8. Expression Change for Transcription

**Figure 8:**
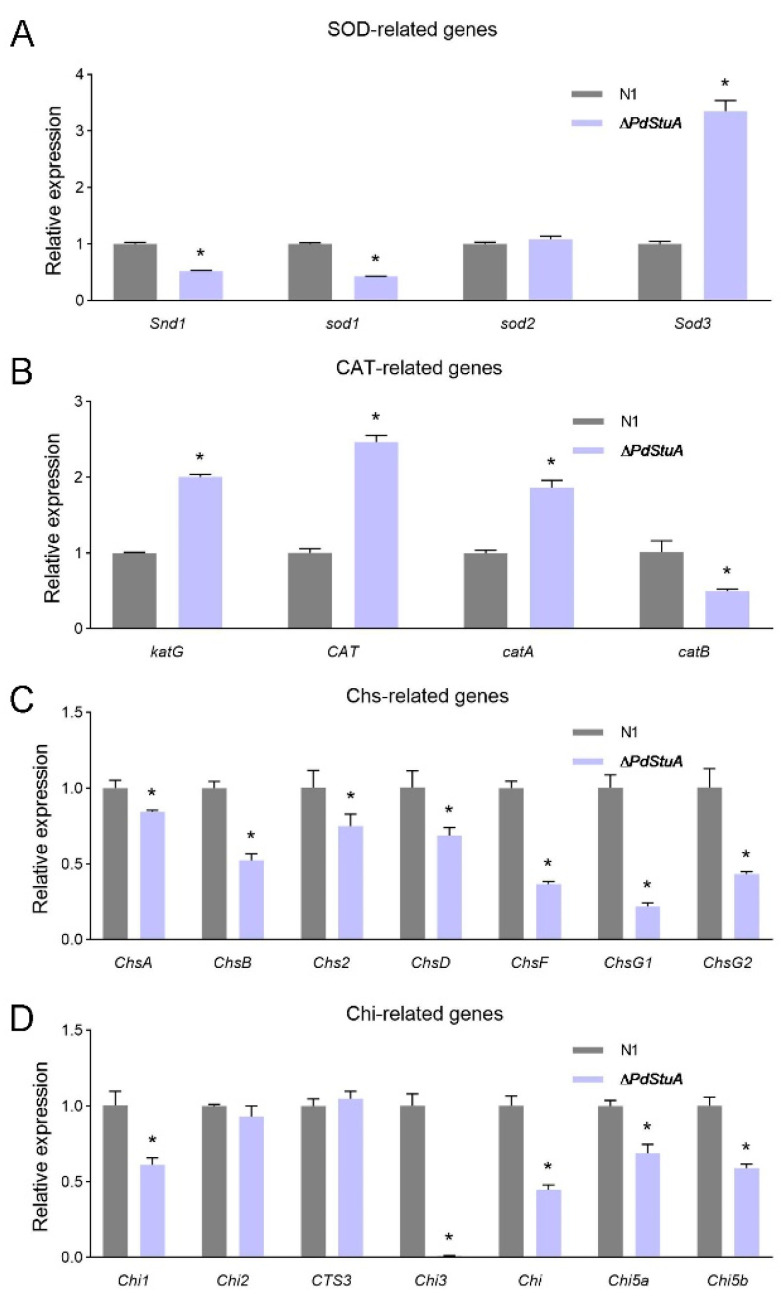
RT-qPCR analysis results of the *PdStuA* gene. The relative expression of SOD (**A**), CAT (**B**), Chs (**C**), and Chi (**D**) genes. The relative mRNA abundance was determined using the 2^−ΔΔCt^ method. * *p* < 0.05.

We assessed the relative expression levels of genes involved in redox reactions and chitin production and degradation based on the stress tolerance assay. Compared to the N1 strain, the expression levels of *Sod3*, *katG*, *CAT*, and *catA* were noticeably elevated, whereas the expression levels of redox-related genes (*Snd1*, *sod1*, and *catB*) were downregulated in the ∆*PdStuA* mutant (Figure 8A,B). The reason for this phenomenon could be that these genes might play different roles in redox reactions. All the chitin synthesis-related genes were downregulated; and, among them, *ChsG1* exhibited the largest reduction (up to 4.6-fold) (Figure 8C). Most chitinase-related genes were significantly downregulated, with *Chi3* being the most severely downregulated and almost not expressed in the ∆*PdStuA* mutant (Figure 8D).

### 3.9. PdStuA Does Not Affect the Pathogenicity of P. digitatum

**Figure 9:**
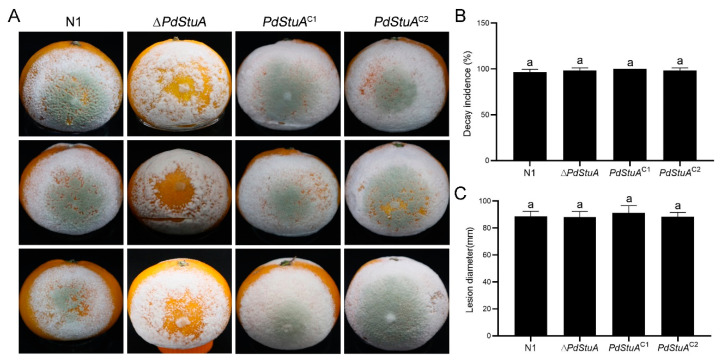
The ∆*PdStuA* mutant does not participate in the virulence of *P. digitatum*. (**A**) Satsuma mandarin fruit was inoculated with 10 μL of spore suspension for testing the N1, ∆*PdStuA*, and *PdStuA*^C1-2^ strains. The photo was taken on Day 4. Statistical analysis of decay incidence (**B**) and lesion diameter (**C**) caused by the four strains. The same letter represents no significant difference, *p* < 0.05.

Infection tests were carried out on citrus fruit to examine the pathogenicity of the N1, ∆*PdStuA*, and *PdStuA*^C1-2^ strains. As shown in Figure 9, the ∆*PdStuA* mutant showed rot symptoms comparable to those of the N1 strain. There was no discernible difference between the N1, ∆*PdStuA*, and *PdStuA*^C1-2^ strains in terms of incidence rate or lesion diameter. However, the symptom of the citrus inoculated with the ∆*PdStuA* mutant was white spores, and there was almost no green spore formation at the inoculation site. We hypothesized that the *PdStuA* gene was not required for the pathogenicity of *P. digitatum* but could reduce the number of spores on the citrus fruit.

## 4. Discussion

APSES protein family members are transcription factors unique to fungi, and they are vital in signal transduction and cellular physiological processes [12,13,14]. Among them, StuA, a transcription factor in the APSES protein family, was the first member discovered and identified [18]. StuA was discovered in *A. nidulans* as a regulator of cell morphogenesis and conidial development as early as 1991 [18]. Previous research has typically shown that the *StuA* gene is primarily responsible for spore and conidiophore development [13,25]. The *StuA* gene, however, has not been investigated in *P. digitatum*. Therefore, we identified a *PdStuA* gene that is highly similar to that of *Aspergillus* (Figure 1). Utilizing homologous recombination, the ∆*PdStuA* mutant was produced (Figure 2). The spore production of the ∆*PdStuA* mutant was dramatically decreased, which was consistent with the results of another study [13,14,15]. However, *PdStuA* did not affect the colony diameter of *P. digitatum*, but previous studies found that deletion of the *StuA* gene could significantly inhibit the diameter of colonies [13,14,15,20]. Therefore, the function of *StuA* was generally conserved among various pathogens, although there were still some minor variations. Consequently, investigation into the *PdStuA* gene is crucial to comprehend the variety of *StuA* gene functions and gain new knowledge about the sporulation mechanism of *P. digitatum*.

A previous study has shown that the central regulatory pathway of the conidium genes in *P. digitatum* were composed of *PdBrlA*, *PdAbaA*, and *PdWetA* [6]. *RodA* and *Som1* are also engaged in spore formation concurrently [9,31]. Previous studies have found a cooperative relationship between the *StuA* gene and *BrlA* as well as *AbaA* in asexual development [12,32,33]. The expression of the hydrophobicity gene *Hyp1* was almost undetectable [15]. Interestingly, the absence of *StuA* resulted in weaker aerial growth and fewer aerial hyphae in *Trichophyton rubrum*, while the results of this study were the opposite [14] (Figure 3). RT-qPCR data showed that deletion of *PdStuA* drastically lowered the expression of the aforementioned genes, with *Aba* and *RodA* showing the greatest reductions in expression (55- and 49-fold, respectively) (Figure 4I). The above experiments demonstrated that the *PdStuA* gene could control the formation of spores and conidiophores by regulating the expression of sporulation-related genes.

RodA is a hydrophobic protein that maintains the stability, permeability, and hydrophobicity of spore surfaces [9,34]. Cell surface hydrophobicity is closely related to pathogenicity. Previous studies have found that disruption of the *FvsuA* and *Hyd1* genes could reduce surface hydrophobicity and pathogenicity [13,35]. Interestingly, deletion of the *PdStuA* gene did cause *P. digitatum* to become less hydrophobic but did not affect its virulence. We speculated that the hydrophobicity genes of *P. digitatum* could not be involved in the pathogenic process, which requires further research.

At present, CFW, CR, and SDS are frequently used to treat fungi and observe their tolerance responses in the study of damage to fungal cell walls [10,36,37,38]. Previous studies have shown that the ∆*AoStuA* mutant exhibits both increased sensitivity to cell wall inhibitors and decreased expression of genes involved in cell wall synthesis [15]. This was consistent with our experimental results. Additionally, we discovered a decrease in the expression of genes involved in the breakdown of the cell wall, with *Chi3* exhibiting the greatest reduction in expression at just 0.01 (Figure 8). The cell wall of ∆*PdStuA* was also observed by TEM to be thicker than that of the N1 strain (Figure 6). All the aforementioned experiments provided evidence that *P. digitatum*’s cell wall might be influenced by the *PdStuA* gene.

According to earlier investigations, peroxisomes and Woronin bodies were detected in the wild type of *A. oligospora* but not in the ∆*AoStuA* mutant [15]. However, this phenomenon was not found in *P. digitatum*. However, ∆*AoStuA* and ∆*PdStuA* mutants were both more sensitive to H_2_O_2_ stress [15]. Additionally, in *F. culmorum*, the sensitivity to oxidative stress was comparable to that in N1 and ∆*FcStuA* mutants, but the activity of catalase was lower in ∆*FcStuA* mutants compared to the N1 strain [22]. In this work, we found that the expression levels of *Snd1* and *Sod1* genes significantly decreased, while the expression levels of the *Sod3* gene significantly increased. Although *Snd1*, *Sod1*, *Sod2*, and *Sod3* all encoded for SOD enzymes, they might play different functions, leading to different levels of gene expression. CAT effectively metabolizes the generated hydrogen peroxide and prevents the harmful effects of hydrogen peroxide accumulation on cells [39]. In this study, the expression levels of *KatG*, *CAT*, and *catA* genes significantly increased, and we speculated that deletion of the *PdStuA* gene could lead to the presence of a large amount of H_2_O_2_ in the mutant, so the genes for clearing hydrogen peroxide were generally upregulated to maintain normal cell life activities.

In summary, this study reveals that *StuA* is an important gene that affects the production of conidia and conidiophores. When the *PdStuA* gene was deleted, conidiophores could not be formed on Day 4, and the growth and hydrophobicity of aerial hyphae were affected. In addition, deletion of the *PdStuA* gene can affect the sensitivity of *P. digitatum* to cell wall inhibitors and H_2_O_2_. Although there was no significant impact on pathogenicity or colony diameter, it can reduce the generation of spores when infecting the host, thereby reducing the spread of green mold disease. Therefore, we believe that these findings can further promote the understanding of the sporulation mechanism of *P. digitatum*.

## Data Availability

The data presented in this study are available on request from the corresponding author. The data are not publicly available at this time as the data also forms part of an ongoing study.

## Supplementary Materials

The following supporting information can be downloaded at: https://www.mdpi.com/article/10.3390/jof9090941/s1, Table S1: Verification primers for deletion vectors, complementation vectors, and mutants. Table S2: RT-qPCR primers used in this study.
