# Supplementary material for: PdStuA Is a Key Transcription Factor Controlling Sporulation, Hydrophobicity, and Stress Tolerance in Penicillium digitatum
Source: J Fungi (Basel). 2023 Sep 18;9(9):941. doi: 10.3390/jof9090941 (PMC10532665; doi:10.3390/jof9090941)
Supplement: Supplementary file 1 [file jof-09-00941-s001.zip › jof-2567228-supplementary.pdf]

Table S1 Verification primers for deletion vectors, complementation vectors, and mutants.

Primer Name	Sequence(5'-3')	Note
PdStuA-DF	CATGCATGGTTGCCTAACTAGGCGCGCCTCGGTCGGAGTTCTCTTT	*PdStuA* deletion
PdStuA-DR	GTAAAACGACGGCCAGTGCCAAGCTTGTGGTCTACGATGTGGTTATG	
PdStuA-UF	ATGACCATGATTACGAATTCGAGCTCAGCCAGAAATAGTATCCCAC	
PdStuA-UR	ATGCGGCTCCACAGCTGCAGGTCGACGCTGGATAGATGACGTTTGA	
*PdStuA*C-F	ATGACCATGATTACGAATTCGAGCTCAGCCAGAAATAGTATCCCAC	*PdStuA* complementation
*PdStuA*C-R	CTCCACAGCTGCAGGTCGACTCTAGAGTAGCAGAAGCAGACGCAAC	
PdStuA-F	TGTGCCTGTCAACGAGCCTA	PCR identification of deletion transformants
PdStuA-R	AGAGTGGGTACAAGAGGTCG	
Hph-F	ATTTGTGTACGCCCGACAGT	
Hph-R	CTCTCGGAGGGCGAAGAATC	
neo-F	ATGATTGAACAAGATGGATTG	
neo-R	GCTCAGAAGAACTCGTCAAGAAG	

Table S2 RT-qPCR primers used in this study.

Gene ID	Gene description	Primer Name	Sequence(5’-3’)	Amplicon size	Note
PDIP_18200	Actin, gamma	*Actin*-F	TCCACTACTGCCGAGCGTGAAAT	218 bp	
		Actin-R	CCGCCAGACTCAAGACCAAGAAC		
PDIP_46840	APSES transcription factor *stuA*	*StuA*-R	ACCATCTGGCTCTCAGCAAC	183 bp	conidiophore-related gene expression
		StuA-F	AGATGGATGGTGCCCACAAG		
PDIP_05330	Regulatory protein *brlA*	*BrlA*-R	CTGCTCAGGTCCAACCATATC	202 bp	
		BrlA-F	AGCTGGAACTCTTCTTGGTAATC		
PDIP_20340	Abacus	*AbaA*-R	TCCTATGGGTCGGAGAAAGT	219 bp	
		AbaA-F	GCTGAGCCATGAACACTAGAT		
PDIP_44350	Developmental regulatory protein *WetA*	*WetA*-F	CCAAAGCAAGAATGGCGTATC	201 bp	
		WetA-R	GATAGAGTCTTGCGTCGTACTG		
PDIP_53340	Hydrophobin, putative	*RodA*-R	GCAACACTGACGTTCGCTTC	136 bp	
		RodA-F	CCAGAGTTGATGTCGGTGGT		
PDIP_56430	CAMP-dependent protein kinase pathway protein (*Som1*)	*Som1*-R	TCTAGCTCGCAACAGCAACA	176 bp	
		Som1-F	ACAGAGCCTTCAAGACGTGG		
PDIP_77720	Catalase-peroxidase 1	*katG*-R	TGTCGGAAGCGAGCCCGAAG	205 bp	CAT-related gene expression
		katG-F	GTTCGCACCAGCGGGACTCT		
PDIP_63740	Catalase	*CAT*-R	CGCCTGGGCGGAAACTACGA	195 bp	
		CAT-F	GATGACCGCGCCAGACCACT		
PDIP_05940	Catalase	*catA*-R	CCGAGATCGAGCAAGCGGCA	200 bp	
		catA-F	GGTTCCCATACCGTCGCGCT		
PDIP_67580	Catalase B	*catB*-R	GCAACGATCTCGCCGTTCGC	202 bp	
		catB-F	GCAGACTGCAACTTCGCGCC		
PDIP_49640	Transcription factor (*Snd1*/*p100*) (Cu/Zn)	*Snd1*-R	CGTGAGGTGGCTACTGTTGT	168 bp	SOD-related gene expression
		Snd1-F	CATTCCACGGCCTTCCTTCT		
PDIP_43140	Superoxide dismutase (Mn)	*sod1*-R	AGACATCGCTGCTCAGATCG	149 bp	
		sod1-F	AGTCAATGGCTTGTGCCAGA		

PDIP_70880	Fe superoxide dismutase (Fe)	*sod2*-R	CGTTCGACTTCTCCTGGACC	159 bp	
		sod2-F	CATCCGCATCGACGGTATCA		
PDIP_66470	Superoxide dismutase (Mn/Inorganic ion transport)	*Sod3*-R	TGAGTGCGTGGTATGTCTCG	151 bp	
		Sod3-F	TCGTCGCCAGCATCAGTATC		
PDIP_07640	Chitin synthase A	*ChsA*-R	GCCGAAAAACTGCCCTCCGC	195 bp	
		ChsA-F	GACGGCACTGCGGAATCCCT		
PDIP_15450	Chitin synthase B	*ChsB*-R	ACGCGCGAGACCGAGTTGTT	206 bp	
		ChsB -F	TTCAGCGTACGCGGGTGGAC		
PDIP_79220	Chitin synthase, putative	*Chs2*-R	TATGCCACAGGCGGGTGCTC	204 bp	
		Chs2-F	GTGCTGGCGGGTCGAGAGAG		
PDIP_26990	Chitin synthase D	*ChsD*-R	CCGCAGGACGTGGAAACCCT	198 bp	Chitin synthase-related gene expression
		ChsD-F	TTCTGCTGGGTGGGTGAGCC		
PDIP_03360	Chitin synthase F	*ChsF*-R	AGTGGTAGCGGGGGAACCCT	192 bp	
		ChsF-F	CTGCCATTCAGGCGGAGGCT		
PDIP_46630	Chitin synthase G	*ChsG1*-R	TCTGTTCAGCAGCAAGCGTA	144 bp	
		ChsG1-F	GTAGCGACGTAGGGAATCGG		
PDIP_24450	Chitin synthase G	*ChsG2*-R	CACCGCCCCTTCCTACCACG	198 bp	
		ChsG2-F	GCATATCTGCGCAAGCCGCC		
PDIP_20480	Chitotriosidase-1	*Chi1*-R	GGAATCTGTGCTCTGGGACC	141 bp	
		Chi1-F	CCCAAGCATGAGCGGTAGAT		
PDIP_53900	Endochitinase 2	*Chi2*-R	AGTTCCAAACCTACCAGCGG	151 bp	
		Chi2-F	AAGAGGAAGGCAAGCCAGTC		
PDIP_53760	Class III chitinase	*CTS3*-R	GGCTTTGACTACCGTGAACT	204 bp	
		CTS3-F	CTTCTCCTGGGACACATAACC		Chitinase-related gene expression
PDIP_48740	Class III chitinase	*Chi3*-R	CCACATCTGCCGAGACAACT	153 bp	
		Chi3-F	GGTCGTTGCCGAGGATAGAG		
PDIP_45890	Chitinase	*Chi*-R	GTATGGCCGAGCGTTTACT	205 bp	
		Chi-F	CGCAATCTCAGGCGTATCA		
PDIP_83360	Class V chitinase	*Chi5a*-R	GCTGCGGCACACAATTATC	202 bp	
		Chi5a-F	CCAAGAAGGATCGTCCGTAAA		

PDIP_29770	Class V chitinase	*Chi5b*-R	GACTGGGTGGTGGTATGTGG	166 bp
		Chi5b-F	TTGGCATACTTGCTCTCGGG	

Note: The following RT-qPCR program was used: 1 cycle of 95 ℃ for 2 min; 40 cycles of 95 ℃ for 15 s, 60 ℃ for 1 min.
